# CATCHU: A NOVEL VISUAL-SOMATOSENSORY INTEGRATION TOOL FOR PREDICTING FALLS

**DOI:** 10.1093/geroni/igad104.1261

**Published:** 2023-12-21

**Authors:** Jeannette Mahoney, Claudene George, Joe Verghese

**Affiliations:** Albert Einstein College of Medicine, Bronx, New York, United States; Montefiore Medical Center, Bronx, New York, United States; Albert Einstein College of Medicine, Bronx, New York, United States

## Abstract

Ability to integrate information across sensory systems is a vital aspect of human functioning in the real world. While multisensory integration effects are measured across a wide array of populations using various sensory pairings and different neuroscience techniques, a gold standard for quantifying multisensory integration was seemingly lacking. We recently established a standardized protocol for administering and calculating behavioral multisensory integration effects across diverse clinical populations and age-ranges. Our previous work has linked the magnitude of visual-somatosensory integration (measured behaviorally using simple reaction time tasks) to important cognitive (attention) and motor (balance, gait, and falls) outcomes in healthy and cognitively impaired older adults. Given the paucity of extant translational multisensory integration tools in clinical settings, we recently developed a novel digital health app grounded in over 15 years of multisensory research. CatchU® (Before You Fall…) quantitatively measures visual-somatosensory integration performance, while considering medical comorbidities, to detect one’s fall propensity. Our goal is to facilitate the identification of patients who are at increased risk of falls and promote physician-initiated falls counseling in routine practice (e.g., annual wellness, sick, or follow-up visits). This will thereby raise awareness of falls and foster physician efforts to alleviate disability, promote independence, and improve the well-being of older adults, all while aiming to reduce the significant societal cost of falls in older adults and others at high risk.

